# Genetic architecture of childhood speech disorder: a review

**DOI:** 10.1038/s41380-024-02409-8

**Published:** 2024-02-16

**Authors:** Angela T. Morgan, David J. Amor, Miya D. St John, Ingrid. E. Scheffer, Michael S. Hildebrand

**Affiliations:** 1 Murdoch Children’s Research Institute, Melbourne, VIC Australia; 2 Speech Pathology, University of Melbourne, Melbourne, VIC Australia; 3 Speech Pathology, Royal Children’s Hospital, Melbourne, VIC Australia; 4 Department of Paediatrics, University of Melbourne, Royal Children’s Hospital, Melbourne, VIC Australia; 5 Epilepsy Research Centre, Austin Health, Melbourne, VIC Australia

**Keywords:** Genetics, Diagnostic markers

## Abstract

Severe speech disorders lead to poor literacy, reduced academic attainment and negative psychosocial outcomes. As early as the 1950s, the familial nature of speech disorders was recognized, implying a genetic basis; but the molecular genetic basis remained unknown. In 2001, investigation of a large three generational family with severe speech disorder, known as childhood apraxia of speech (CAS), revealed the first causative gene; *FOXP2*. A long hiatus then followed for CAS candidate genes, but in the past three years, genetic analysis of cohorts ascertained for CAS have revealed over 30 causative genes. A total of 36 pathogenic variants have been identified from 122 cases across 3 cohorts in this nascent field. All genes identified have been in coding regions to date, with no apparent benefit at this stage for WGS over WES in identifying monogenic conditions associated with CAS. Hence current findings suggest a remarkable one in three children have a genetic variant that explains their CAS, with significant genetic heterogeneity emerging. Around half of the candidate genes identified are currently supported by medium (6 genes) to strong (9 genes) evidence supporting the association between the gene and CAS. Despite genetic heterogeneity; many implicated proteins functionally converge on pathways involved in chromatin modification or transcriptional regulation, opening the door to precision diagnosis and therapies. Most of the new candidate genes for CAS are associated with previously described neurodevelopmental conditions that include intellectual disability, autism and epilepsy; broadening the phenotypic spectrum to a distinctly milder presentation defined by primary speech disorder in the setting of normal intellect. Insights into the genetic bases of CAS, a severe, rare speech disorder, are yet to translate to understanding the heritability of more common, typically milder forms of speech or language impairment such as stuttering or phonological disorder. These disorders likely follow complex inheritance with polygenic contributions in many cases, rather than the monogenic patterns that underly one-third of patients with CAS. Clinical genetic testing for should now be implemented for individuals with CAS, given its high diagnostic rate, which parallels many other neurodevelopmental disorders where this testing is already standard of care. The shared mechanisms implicated by gene discovery for CAS highlight potential new targets for future precision therapies.

## Introduction

Speech acquisition is a biologically driven, inexorable developmental process in most infants. Yet up to 5% of children develop common speech disorders including stuttering, articulation and phonological impairments [Table 1]. These common conditions are highly tractable and tend to resolve, with or without intervention, by 7 years of age [1, 2]. By contrast, 1 in 1000 children follow a severely disrupted developmental path to an intractable speech disorder known as childhood apraxia of speech (CAS) [3]. In these individuals, early development is often marked by hypotonia, feeding difficulties, limited babbling, delayed onset of first words, and marked difficulty in acquiring speech which is unintelligible in the preschool years, when a diagnosis is usually made [4]. The condition was first described by pioneering British speech therapist Muriel Morley in 1957 who identified a childhood speech presentation akin to the speech praxis seen in adults following lesions to Broca’s area, with the crux of the diagnosis being difficulty accurately producing sound sequences [5].

**Table 1:** Speech disorder phenotypes.

Speech disorder	Operational definition	Prevalence	Natural history & tractability	Aetiology
Articulation [39]	Disorder of speech sound production. Consistently distorts one or more speech sounds (phones) in absence of known cause (e.g., hearing loss, cleft palate, missing teeth). Prosody unaffected.	5% preschoolers	Highly tractable, majority resolve by 7 years^	Complex multifactorial
Phonological [39]	Disorder in understanding/use of speech sounds (phonemes) of language to convey meaning. Child makes atypical errors seen in <10% of peers, e.g. phonological process of “backing”, where a posteriorly produced sound is used in place of an anteriorly produced sound, e.g., says key for tea, or gog for dog. Vowels, prosody unaffected.	5% preschoolers^	Highly tractable, majority resolve by 7 years	Complex multifactorial
Stuttering [40]	Disorder of speech fluency characterized by repetitions (of sounds, syllables, words and/or phrases), prolongation of sounds, and hesitations and/or blocks.	10% preschoolers	Tractable in some, 65% developmental forms resolve by 7 years	Monogenic, complex multifactorial
Dysarthria [13]	Disorder of central or peripheral nervous system affecting neuromuscular control and tone, e.g., spasticity, ataxia, fluctuating tone, involuntary movements. This results in imprecision of speech due to impairments in one or more areas of phonation, articulation, prosody, resonance.	0.1% preschoolers	Less tractable, never resolves but responsive to therapy	Monogenic, complex multifactorial
Childhood apraxia of speech (CAS) [7]	Disorder of motor programming/planning. Core features: 1. inconsistent production of consonants and vowels across repeated productions, 2. lengthened and impaired coarticulatory transitions between sounds and syllables (e.g., omissions of sounds, vowel errors, repetitions), 3. inappropriate prosody/disrupted intonation, e.g., placing stress on a typically unstressed syllable or using equal stress across all syllables.	0.1% preschoolers	Less tractable, rarely resolves but responsive to therapy	Monogenic, complex multifactorial

Table 1 key: focuses on neurodevelopmental forms of speech disorder, not structural (eg. cleft lip or palate, malocclusion of mandible and maxilla; or acquired (eg. brain tumour, stroke, traumatic brain injury). *Some children have phonological *delay* as opposed to *disorder*. This is a delay, in understanding/use of speech sounds of one’s language to convey meaning. A child persists in the use of developmental error patterns as seen in the phonology of younger children, eg. a 6 year old using the phonological process of stopping fricatives, substituting a ‘b’ for ‘f’ (bish for fish), which should have resolved at age 4 years. Vowels and prosody are unaffected.

Since the original description of CAS, there has been ongoing debate over the defining diagnostic features of the condition [6]. In 2007, the American Speech and Hearing Association supported an expert-based consensus which defined the three diagnostic features of CAS (Table 1) [7]. Whilst the condition is largely framed as a ‘motor’ speech disorder resulting from movement planning or programming deficits, language and literacy impairments also occur in over 90% of individuals [8–10]. Furthermore, neuroimaging points to perturbation of linguistic as well as motor pathways, in affected individuals [11].

Recently, the CAS phenotype has increasingly been associated with commonly occurring neurodevelopmental comorbidities, including motor and cognitive impairments, attention deficit hyperactivity disorder, seizures and autism spectrum disorders [8–10]. Similar to the presentation of these neurodevelopmental disorders (NDDs), speech and language disorders rarely occur in isolation, and rather are found in a broader context of perturbed neurodevelopment.

Until recently, understanding of the aetiology of CAS was limited. Parents of children with CAS would embark on a diagnostic odyssey to investigate the chronic and striking nature of the condition. Early studies have implicated copy number variants (CNVs), including chromosomal aneuplodies involving multiple genes, and single nucleotide variants (SNVs) in individual genes, to CAS.

A specific neurogenetic basis for CAS was first identified in 2001, with the seminal discovery that pathogenic missense SNVs in *FOXP2* [12], a transcriptional repressor, were associated with CAS, initially inherited in a large multiplex family, but subsequently also found to arise de novo in sporadic cases. Functionally related transcription factors and downstream targets of *FOXP2* were subsequently investigated, namely *CNTNAP2* (MIM: 604569), *FOXP1* (MIM: 605515) and *TBR1* (MIM:606053). Although these genes have been associated with intellectual disability syndromes and ASD, they have not explained cases ascertained for primary or isolated speech or language disorder [13–15]. The next most promising candidate gene for CAS was *GRIN2A*, which is also associated with the epilepsy-aphasia syndromes, now termed developmental and/or epileptic encephalopathy with spike-wave activation in sleep [16], and including Llandau-Kleffner syndrome [17–19]. Yet again, as for *CNTNAP2*, *FOXP1* and *TBR1*, pathogenic variants in *GRIN2A* have not been identified in cohorts ascertained for a primary diagnosis of speech or language disorder in the absence of epilepsy.

Advances in microarray technology have also led to numerous chromosomal deletions being associated with CAS, but typically in the presence of cognitive impairment or ASD, such as 16p11.2 deletion [20, 21]. Some of these CNVs have drawn attention to possible genes in the pathogenesis of CAS such as 18q12.3 microdeletions encompassing *SETBP1* [22], 12p13.33 microdeletions including *ELKS/ERC1* [23], 2p15-p16.1 microdeletions encompassing and proximal to *BCL11A* [24], 7q11.23 duplication syndrome [25] implicating a number of genes and 17q21.31 deletion or Koolen-de Vries syndrome encompassing *KANSL1* [26].

Most recently, advances in massively parallel sequencing technologies and bioinformatic algorithms have allowed rapid identification of genes not previously implicated in speech dysfunction. Here we review the rapidly unfolding Mendelian genetic bases for CAS. Specifically, we have reviewed data on gene discovery cohorts applying exome or genome sequencing to cohorts ascertained for primary speech disorder CAS [Search strategy box below].

## Search strategy and selection criteria

We searched PubMed for articles published between Jan 1, 2001, and March 15, 2023, using the search terms “childhood apraxia of speech”, “dyspraxia”, “speech”, “exome sequencing” and “genome sequencing”. There were no language restrictions. We selected articles that had ascertained cohorts with CAS and applied next generation sequencing approaches and analysis to report novel genes associated with CAS. We also searched for articles describing the function and implications of pathogenic variants in the genes identified, for literature on other neurodevelopmental disorders associated with these genes. The final reference list was generated based on the relevance to the topics covered in this review.

## Discussion/analysis of recent literature

**Fig. 1:**
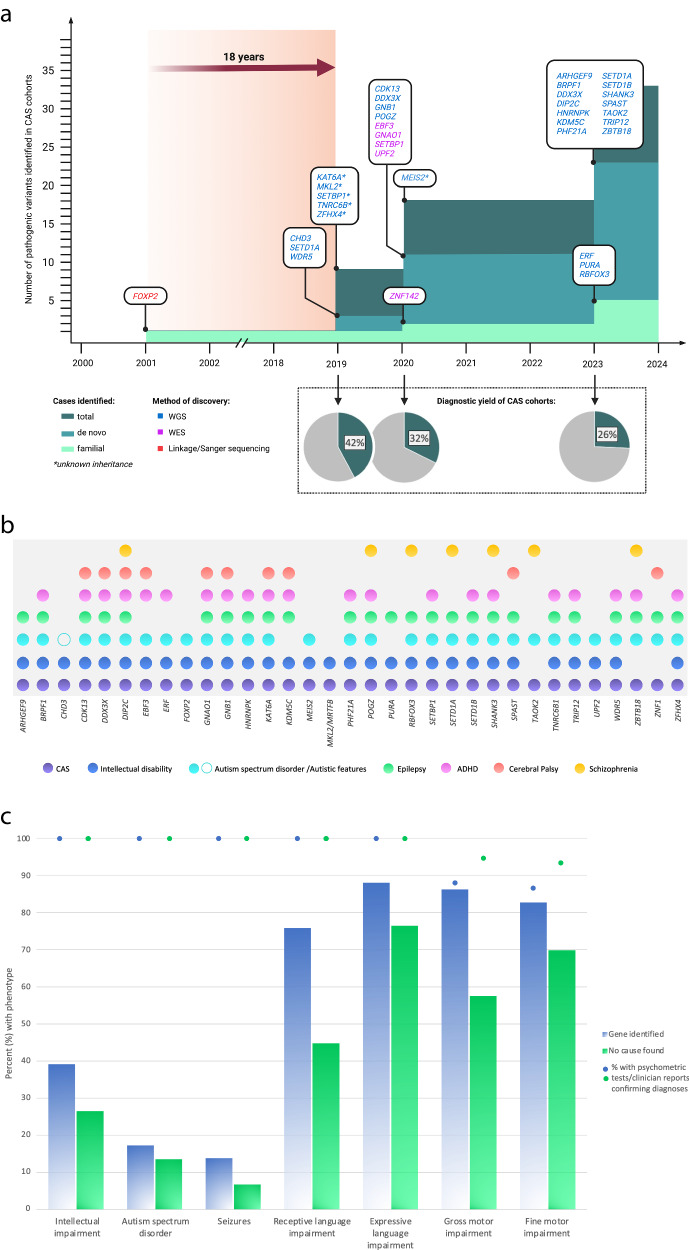
Genetic causes for childhood apraxia of speech over time. **a** Genetic causes identified for CAS over time. WGS, whole genome sequencing; WES, whole exome sequencing. More patients have undergone WGS to date. The yield to date has been similar for WGS and WES. No variants have been reported in non-coding regions to date. **b** CAS candidate genes and co-occurring neurodevelopmental phenotypes. An association between a gene and a phenotype was denoted if the association was higher than the prevalence in the general population. **c** Overlap in phenotype in children with CAS, comparing those with a pathogenic variant and those with no known cause. Phenotypic features of CAS cohorts with (*n* = 29) and without (*n* = 74) pathogenic genetic variants, based on data from Hildebrand et al. (2020) and Kaspi et al. (2023). Authors were approached for any updated diagnoses for study participants. Confirmation of diagnoses for cognitive impairment, ASD, receptive and expressive language impairment, and gross and fine motor impairment was based on psychometric testing/clinical report. Seizure diagnosis is based on parent report.

Three CAS gene discovery cohort studies were identified, each relatively small given the rarity of the disorder, but growing in cohort size over time: *n* = 19 probands, Eising et al., 2019; *n* = 33 probands, Hildebrand et al., 2020; *n* = 70 probands, and Kaspi et al., 2023. In the first study, 8/19 (~42%) probands were found to have a pathogenic or likely pathogenic gene variant via genome sequencing [8]. In the second study, 11/34 (~32%) probands had highly plausible pathogenic variants identified by a combination of exome and genome sequencing, and chromosomal microarray analysis [9]. In the third study, 18/70 (~26%) probands had a high confidence pathogenic variant detected via genome sequencing or chromosomal microarray analysis [10]. There was no apparent benefit at this stage for WGS over WES in identifying monogenic conditions associated with CAS. The overall clinical genetic diagnostic yield across the three cohorts was 30% (36/122 probands) (see Fig. 1a).

These studies provided the first neurobiological insights into the mechanisms of speech disorders, including the key finding that pathogenic variants are enriched in genes involved in transcriptional regulation and chromatin remodelling in the developing brain (Table 2). Importantly, these genes also showed significant clustering within a module of genes highly co-expressed in the human embryonic brain, in regions known to subserve speech function [8]. Hence the speech disorders field now has the first evidence that CAS is a neurodevelopmental disorder due to dysregulation of genes expressed in white-matter tracts critical for development of speech [8, 9, 27].

**Table 2:** Genes causally linked to CAS and other neurodevelopmental phenotypes between 2001 and 2023.

Gene	Disease name, OMIM	Loci	Mode of inheritance	Year, method of discovery	Molecular pathway & function	Associated features	Independent cases linked to CAS	Strength of evidence
*FOXP2*	*FOXP2*-Related Speech & Language Disorder, 605317	7q31.1	AD, de novo	2001, Linkage/Sanger sequencing	Transcriptional regulation. Encodes putative forkhead box P2 transcription factor with forkhead DNA binding domain and polyglutamine tract [12]. Expressed in foetal and adult brain in regions important for speech and language development. Regulates at least 34 target genes in developing human brain including neurodevelopmental genes *CNTNAP2*, *FOXP1, TBR1* [41].	Cognition ranges from average to mild ID, feeding difficulties in infancy, fine & gross motor impairment, ASD, language impairment, anxiety, depression, sleep disturbance.	Morison et al., 2023 [29]	High
*CHD3*	Snijders Blok-Campeau syndrome, 618205	17p13.1	AD, de novo	2019, WGS	Chromatin-remodeller/reader. Member of CHD family of ATP-dependent chromatin remodelling proteins that modulate gene expression. Encodes chromodomain DNA helicase ATPase highly expressed during early brain development. Remodels chromatin through deacetylation of histone proteins to modulate downstream transcription factors important for specification of cortical layering [42].	ID/DD, macrocephaly, prominent forehead, hypertelorism, hypotonia, joint laxity, severity of neurologic deficits & presence of non-neurologic features are variable. Autistic features are commonly reported.	Snijders Blok et al., 2018 [43]	Medium
*KAT6A*	*KAT6A* syndrome, 601408	8p11	AD, de novo	2019, WGS	Chromatin writer/reader. Encodes for member of MYST family of histone methyltransferases, with wide range of core cellular functions, e.g., chromatin remodelling, transcriptional regulation, protein translation, metabolism, cellular replication. Forms complex with *KAT6B*, *BRPF1*, and two non-catalytic subunits with role in development of neural and hematopoietic stem cells [44].	ID, vision impairment, GI dysfunction, sleep disturbance, ASD, majority minimally verbal & rely on alternate communication. Rates of epilepsy, ADHD, CP higher than typical population.	St John et al., 2022 [45]	High
*MKL2/MRTFB^*	*MRTFB*-related disorder, 609463	16p13.12	De novo	2019, WGS	Transcriptional regulation. This transcriptional regulator binds with its cofactor, *serum response factor* (*SRF)* to regulate over 300 genes, including those that regulate the actin cytoskeleton of cells critical for development (*ACTB*, *ACTG1*, *GSN*) and synaptic activity (*CDK5R1*, *CDK5*, *RYR1*, *RYR3*, *CLTC*, *DLG4*, *ARC*) [46].	ID, GDD, CAS, mild dysmorphic features, impulse control issues.	Andrews et al., 2023 [46]	High
*SETBP1*	*SETBP1* haplo-insufficiency disorder, 616078	18q12.3	AD, de novo	2019, WES	Transcriptional activity and expression. SETBP1 binds to gDNA in AT-rich promoter regions, causing activation of gene expression through recruitment of *HCF1/KMT2A/PHF8* epigenetic complex. Perturbed binding of *SETBP1* to gDNA impairs target gene upregulation. *SETBP1* target genes are key controllers of brain morphogenesis as shown by *in utero* brain electroporation of mutated *SETBP1* which impairs mouse neurogenesis leading to profound delay in neuronal migration [47].	ID, mild motor disorder, hypotonia, ASD, ADHD, vision impairment (refractive errors & strabismus).	Morgan et al., 2021 [30]	High
*SETD1A*	*SETD1A* neuro-developmental disorder, 619056	16p11.2	AD, de novo	2019, WGS	Transcriptional regulation/ epigenetic writer (histone methylation). *SETD1A*-mediated H3K4me3 is important for regulating cell cycle (e.g., activates β-catenin expression, required for proliferation of neuronal progenitor cells) and neuronal processes underlying normal cognitive functioning [48].	ID/DD, epilepsy, facial dysmorphism subtle, increased risk of ASD and schizophrenia.	Kummeling et al., 202 [48]	Medium
*TNRC6B*	*TNRC6B*-related syndrome, 610740	22q13.1	AD, de novo	2019, WGS	Transcriptional regulation and RNA binding. Encodes for one of three paralogue proteins, involved in translational inhibition. *TNRC6A, TNRC6B*, and *TNRC6C* associate with Argonaute family of proteins to coordinate posttranscriptional gene silencing [49].	ID/DD, fine & gross motor delay, ASD, ADHD, musculoskeletal findings. Rate of epilepsy higher than in the general population.	N/A	Low
*WDR5*	*WDR5* neuro-developmental disorder, 609012	9q34.2	AD, de novo	2019, WGS	Transcriptional regulation/chromatin scaffolding. Encodes a member of the WD repeat protein family. Members of this family are involved in variety of cellular processes and chromatin scaffolding functions including cell cycle progression, signal transduction, apoptosis, and transcriptional regulation [50].	ID, speech & language impairments, epilepsy, ASD, ADHD.	Snijders Blok et al., 2023 [51]	Medium
*ZFHX4*	*ZFHX4*-associated syndrome, 606940	8q21.11	AD, de novo	2019, WGS	Transcriptional regulation. Encodes for transcription factor important in embryonic processes, including regulating neural and mesenchymal cell differentiation. ZFHX4 binds to and modulates function in nucleosome remodelling and deacetylation [52].	ID, hypotonia, sleep issues, over-friendly, anxiety, ASD, ADHD, imbalance, seizures/epilepsy.	N/A	Low
*CDK13*	*CDK13*-related disorder, 603309	7p14	AD, de novo	2020, WGS	Transcriptional regulation/expression. Encodes a member of ATP-dependent serine/threonine protein kinase family important in cell cycle control, transcription regulation, mRNA processing and hematopoiesis via phosphorylation [53].	ID/DD, recognizable facial features, behavioral findings, feeding difficulties in infancy, structural cardiac defects, seizures, ASD, ADHD. Reports of CP.	Morison et al., 2023 [54]	High
*DDX3X*	*DDX3X* syndrome, 300160	Xp11.3–11.23	X-Linked	2020, WGS	Transcriptional regulation. Encodes for member of conserved DEAD-box protein family. This family has ATP-dependent RNA helicase activity, and is important for transcription regulation, gene splicing, RNA transport in the nucleus, translation, cell signalling and viral replication in the cytoplasm. *DDX3X* has important roles in cell cycle control, apoptosis, and tumorigenesis [55].	ID/DD, hypotonia, feeding difficulty in infancy, ASD, ADHD, self-injurious behaviour, poor impulse control, aggression, many affected females remain nonverbal after age 5 years. Reports of epilepsy, CP.	N/A	Medium*
*EBF3*	*EBF3* neuro-developmental disorder, 607407	10q26.3	AD, de novo	2020, WES	Transcriptional regulator of neurogenesis, differentiation. Encodes for member of family of highly homologous transcription factors. Roles in B-cell differentiation, bone development, neurogenesis, laminar formation of cerebral cortex. *EBF3* is a downstream transcriptional target of *ARX* and thought to be repressed by *ARX* [56].	ID/DD, speech delay, gait or truncal ataxia/CP, hypotonia, behavioural problems, facial dysmorphism, significant variability between individuals. Rates of ASD/ADHD higher than in typical population.	Chao et al., 2017 [57]	High
*GNAO1*	*GNAO1* encephalopathy, 139311	16q13	AD, de novo	2020, WES	Synaptic protein/signal transduction. Encodes a guanine nucleotide-binding protein alpha subunit; part of family of signal-transducing molecules. Most abundant membrane protein in mammalian central nervous system, constituting 1% of total brain membrane protein [58].	ID/DD, early infantile seizures, involuntary movements, CP. Rates of ASD/ADHD higher than in typical population.	Wirth et al., 2020 [59]	Unclear
*GNB1*	*GNB1* encephalopathy, 139380	1p36.33	AD, de novo	2020, WGS	Synaptic protein/signal transduction. Encodes a guanine nucleotide-binding protein beta subunit, which is part of a large family of signal-transducing molecules [60].	ID/DD, structural brain anomalies, infantile hypotonia, CP, seizures. Rates of ASD/ADHD higher than in typical population.	N/A	Low
*MEIS2*	*MEIS2*-related condition, 601740	15q14	AD, de novo	2020, WGS	Transcriptional regulation. Encodes for *MEIS2* homeobox protein, which belongs to TALE homeodomain transcription factor family (alongside *MEIS2* and *MEIS3*). Roles in cell migration, apoptosis, and metabolism [61].	ID, facial dysmorphology, ASD, cleft palate, cardiac septal anomalies.	Douglas et al., 2018 [62]	High
*POGZ*	White-Sutton syndrome (*POGZ*-related disorder), 614787	1q21.3	AD, de novo	2020, WGS	Transcriptional regulation/chromatin-related. Encodes for a zinc-finger protein found in the cell nucleus. *POGZ* influences chromatin remodelling by binding to chromatin, and impacting gene transcription [63].	ID/DD (wide spectrum of cognitive dysfunction), hypotonia, epilepsy, ASD, ADHD, behavioral issues. Schizophrenia reported.	Nagy et al., 2022 [64]	Unclear
*UPF2*	*UPF2*-related disorder, 605529	10p14	AD, de novo	2020, WES	Transcriptional regulation. Encodes a core factor of the nonsense-mediated mRNA decay (NMD) complex alongside *UPF3B* and *UPF1* that regulates transcription [65].	ID, ASD, speech disorder.	N/A	Low
*ZNF142*	*ZNF142*-related neuro-developmental disorder, 604083	2q35	AR	2020, WES	Transcriptional regulation. Encode member of Kruppel family of C2H2-type zinc finger proteins, involved in transcriptional regulation, signal transduction, meiotic recombination, DNA repair, cell proliferation and differentiation [66].	ID, variable manifestation of seizures, tremor, dystonia.	Khan et al., 2019 [67]; Christensen et al., 2022 [68]	Medium
*ARHGEF9*	*ARHGEF9*-related disorder & encephalopathy, 300429	Xq11.1	X-linked	2022, WGS	Synaptic protein/signal transduction. Encodes Rho guanine nucleotide exchange factor that connects microtubule and actin cytoskeleton dynamics and is required for mitotic spindle formation and orientation that is critical for synaptic function [69].	ID, epilepsy, ASD, dysmorphology.	N/A	Low
*BRPF1*	*BRPF1*-related disorder, 602410	3p25	AD, de novo	2022, WGS	Chromatin reader/writer. Forms complex with *KAT6A* to activate histone acetylation. Role in transcriptional regulation, chromatin binding and remodelling [70].	ID, facial dysmorphology, ptosis, variable expressivity speech & language disorder. Rates of ASD/ADHD higher than in typical population.	Yan et al., 2017 [71]	Medium
*DIP2C*	*DIP2C*-related disorder, 611380	10p15.3	AD, de novo	2022, WGS	Transcriptional regulation. Encodes member of disco-interacting protein homolog 2 family, with important roles in cell growth, cell cycle regulation, and migration related to DNA methylation and gene expression changes [72].	ID, ASD, speech & language disorder. Reports of epilepsy, schizophrenia, CP.	N/A	Low
*ERF*	*ERF*-related craniosynostosis, 611888	19q13	AD, de novo	2022, WGS	Transcriptional regulation. Encodes for member of ETS family of transcription factors, which regulates cell proliferation and differentiation.	ID, ASD, ADHD, craniosynostosis (often postnatal), visual impairment, facial dysmorphism, speech delay, poor gross & fine motor control, hyperactivity, poor concentration.	N/A	Low
*HNRNPK*	*HNRNPK*-related condition, 600712	9q21.32	AD, de novo	2022, WGS	Transcriptional regulation. Encodes for conserved RNA-binding protein, involved in gene expression (chromatin remodelling and gene transcription), mRNA splicing, translation, and stability [73].	ID/DD, motor delay, speech delay, structural brain abnormalities, epilepsy, ASD, ADHD, dysmorphic features, hypotonia, skeletal abnormalities, hand/feet abnormalities, cardiac abnormalities, genitourinary issues.	N/A	Low
*KDM5C*	*KDM5C* neurodevelopmental disorder, 314690	Xp11.22	X-linked, de novo	2022, WGS	Chromatin eraser/reader. Encodes a histone demethylase protein that functions as a transcriptional repressor through the REST complex [74].	ID, short stature, facial dysmorphology, epilepsy, ADHD, behavioural disorders, spasticity/CP.	Jensen et al., 2005 [75]; Tzschach et al., 2006 [76]; Leonardi et al., 2023 [77]	Unclear
*PHF21A*	*PHF21A*-related condition, 608325	11p11.2	AD, de novo	2022, WGS	Transcriptional regulation/chromatin reader. Encodes for a protein that binds to demethylated histones to repress gene expression [78].	ID, hypotonia, dysmorphic features, speech & language delay, ASD, ADHD, epilepsy.	N/A	Low
*PURA*	*PURA* syndrome, 600473	5q31	AD, de novo	2022, WGS	Transcriptional regulation. Encodes for a highly conserved Pur-alpha protein, involved in DNA and RNA binding, contributing to transcription regulation, DNA replication, RNA transport and mRNA translation. Important for neurogenesis, synapse formation, and myelin maturation [79].	ID, severely delayed walking & motor skills (may not walk), hypotonia, dysphagia, epilepsy, heart abnormalities, urogenital, respiratory, GI & skeletal anomalies, hormone disorders, minimally verbal or no speech.	N/A	Low
*RBFOX3*	*RBFOX3*-related disorder, 616999	17q25.3	AD, de novo	2022, WGS	Transcriptional regulation. Encodes for member of RNA-binding Fox protein family that regulates RNA splicing. *RBFOX3* is exclusively expressed in neurons and critical in brain development via regulation of neuronal differentiation, hippocampal neurogenesis, synaptogenesis [80].	ID, epilepsy, ASD, speech & language impairment. Schizophrenia reported.	Lal et al., 2013 [81]	High
*SETD1B*	*SETD1B*-related neurodevelopmental disorder, 611055	12q24.31	AD, de novo	2022, WGS	Transcriptional regulation/chromatin-related epigenetic writer (histone methylation). Encodes lysine-specific catalytic SET domain protein of histone methyltransferase complex. Plays important role in epigenetic regulation of gene transcription. Member of COMPASS complex including *KMT2A-D and KMT2F* [82].	ID/DD (precedes seizure onset), ASD, ADHD, variable epilepsy phenotypes, behavioural issues; males over-represented.	N/A	Low
*SHANK3*	*SHANK3*-related condition, 606230	22q13	AD, de novo	2022, WGS	Synaptic protein. Encodes for scaffolding protein enriched in postsynaptic densities of excitatory synapses required for formation and maturation of dendritic spines [83].	ID/DD, ASD, ADHD, epilepsy, absent to severely delayed speech. Schizophrenia reported.	Brignell et al., 2021 [84]	High^#^
*SPAST*	Spastic paraplegia type 4, 604277	2p22.3	AD, de novo	2022, WGS	Encodes for protein, spastin, which is a microtubule-severing ATPase and member of AAA protein family. Role in regulating microtubule cytoskeleton length, structure; important in organelle transport, cell division, neuronal morphogenesis [85].	ID/DD, motor & speech delay, ASD, progressive ascending spasticity/CP, dystonia, neurogenic bladder dysfunction, GI dysmotility, epilepsy.	N/A	Low
*TAOK2*	*TAOK2* neurodevelopmental disorder, 613199	16p11.2	AD, de novo	2022, WGS	Signal transduction. Encodes for serine/threonine protein kinase involved in cell signalling, microtubule organization, stability, and apoptosis.	ASD, speech & language impairments. At risk for schizophrenia.	N/A	Low^‡^
*TRIP12*	*TRIP12*-related condition, 604506	2q36.3	AD, de novo	2022, WGS	Transcriptional regulation. Encodes for a chromatin remodelling E3 ubiquitin-protein ligase, important in cell cycle progression and maintenance of genome integrity. Important in DNA replication, mitotic progression, chromosome stability [86].	ID/DD, ASD, epilepsy, speech & language disorder. Rate of ADHD higher than typical population.	N/A	Low
*ZBTB18*	*ZBTB18*-related condition, 608433	1q44	AD, de novo	2022, WGS	Transcriptional regulation. Encodes a transcriptional repressor of key pro-neurogenic genes. Involved in chromatin assembly [87].	ID, hypotonia, microcephaly, corpus callosal anomalies, epilepsy, ADHD, growth problems, variable facial dysmorphologies, speech & language impairments.	N/A	Low

Table 2 key: ^*MKL2* now known as *MRTFB*, *AD* autosomal dominant, *AR* autosomal recessive, *ID* intellectual disability, *DD* developmental delay, *ASD* autism spectrum disorder, *ADHD* attention deficit hyperactivity disorder, *CP* cerebral palsy, *GI* gastrointestinal.

Strength of evidence: Low = single case in one CAS cohort; Medium = >1 case, <20% verbal cases in an independent reverse phenotyping study; High = >20% of verbal cases in an independent phenotyping study.

Unclear = independent study reporting severe speech disorder but no assessment of CAS.

*Independent cases found in Hildebrand et al. (2020) and Kaspi et al. (2022) cohorts.

^#^2/3 with CAS in a cohort of individuals with Phelan-McDermid/22q13 deletion syndrome, caused by heterozygous loss of function of *SHANK3*.

^‡^34/44 (77%) individuals with CAS in a cohort of individuals with 16p11.2 deletion syndrome, of which *TAOK2* is a core gene (Mei et al., 2018).

Unlike *FOXP2*, which had no disease association prior to being linked to CAS, many newer genes associated with CAS were already implicated in other NDDs such as intellectual disability (ID), ASD and epilepsy (see Table 2 for outline of known associated conditions). These findings [8–10] align with the well-established genetic overlap between other neurodevelopmental phenotypes [28] and indicate that CAS can be added to these overlapping profiles [see Fig. 1b].

In some ways, the association of CAS with genes known to cause ID, ASD and epilepsy is not surprising given that these neurodevelopmental phenotypes have long been associated with speech and language pathology; however, a primary speech phenotype of CAS had been considered separate from the larger group of NDDs. Now genetic findings show this distinction may not be valid. Furthermore, recent studies of individuals with *FOXP2* variants show that they also experience broader, subtle, neurodevelopmental phenotypes beyond speech dysfunction [29]. Thus, whilst *FOXP2* remains the most ‘speech specific’ gene to be identified [29], there may not exist a “pure” speech apraxia gene. As such, speech and neurodevelopmental phenotypes should be considered as existing across a phenotypic spectrum rather than as categorical diagnoses, mirroring findings in genetic understanding of other diseases, such as epilepsy.

Finally, there is currently a strong bias in comparing next generation sequencing findings for ID, ASD and epilepsy, with tens of thousands of probands reported in the literature, compared to just over 120 probands with CAS. Thus, surprisingly, the published CAS cohort studies have shown a comparably high genetic diagnostic yield for individuals with these speech phenotypes, despite them arguably being milder relative to ID, ASD and epilepsy. This suggests clinical genetic testing is also warranted for children with CAS given that genome-wide testing is increasingly routine and often funded for children with other NDDs. Routine clinical genetic testing will be important as although many of the gene variants reported to date are de novo and predicted pathogenic according to ACMG guidelines, most have been found only in individual probands and identification of the same gene in unrelated patients with the same phenotype will confirm that gene’s contribution. As outlined below, unrelated patients have been identified for three of the candidate genes for CAS across the small CAS-ascertained cohort studies alone.

Although genetic heterogeneity is a feature of gene discovery findings in CAS cohorts, pathogenic variants in a handful of genes, namely *SETBP1*, *SETD1A* and *DDX3X*, each account for multiple cases across the cohorts studied to date [8–10]. *SETBP1* stands out as being particularly intriguing, with pathogenic loss-of-function (LoF) variants detected in all three cohorts [8–10] With emerging evidence for CAS in *SETBP1* haploinsufficiency disorder, a speech and language study of 28 individuals with *SETBP1* LoF variants then confirmed the diagnosis of CAS, seen in 80% of individuals studied, as a core part of the phenotype [30]. When comparing children’s performance across developmental domains, it was also clear that communication was most impaired relative to social skills, daily living skills, motor abilities and adaptive functioning, supporting *SETBP1* having a central role in speech and language development [30]. Further, studies of common genetic variants suggest *SETBP1* may also be important for communication abilities in the general population. Associations between single nucleotide polymorphisms (SNPs) in *SETBP1* and scores on a test examining syntactic complexity were reported in a genome wide association study of language disorder in a geographically isolated Russian cohort aged 3–18 years [31]. SNPs in *SETBP1* have also been associated with phonological working memory in a reading-impaired cohort [32].

In addition to evidence for the strength of association between CAS and the candidate genes across the three CAS-ascertained gene discovery cohorts discussed here, Table 2 further outlines the strength of independent evidence currently found to support the candidate genes. At this time, nine of the candidate genes have a high level of independent supporting evidence *(FOXP2, KAT6A, MKL2/MRTFB, SETBP1, CDK13, EBF3, MEIS2, RBFOX3, SHANK3)*, six have medium *(CHD3, SETD1A, WDR5, DDX3X, ZNF142, BRPF1)* and the remainder have low levels of independent evidence, but we expect expanded clinical genetic testing will reveal additional cases for many of the other candidate genes implicated [8–10].

### Alternative genetic mechanisms for CAS

If high impact de novo sequence variants and CNVs account for about one third of individuals with CAS, the question that follows is what genes or mechanisms account for the remaining unsolved cases. Whole genome sequencing has not been completed for all cases studied, meaning non-coding variants have not been routinely interrogated and may account for some undiagnosed cases. Mosaicism, increasingly implicated in neurodevelopmental diseases such as intellectual disability, epilepsy and autism [33], may be low level and brain-limited and may underpin CAS in some individuals where it may be limited to key networks; however, detection would require sequencing of brain tissue, which is generally inaccessible.

From existing data, there is evidence that the cohort of CAS individuals with an identified pathogenic de novo gene variant is enriched for individuals with cognitive impairment and co-morbid language and motor diagnoses, compared to those without a genetic diagnosis (Fig. 1c). These data suggest that different genetic mechanisms may apply to those cases with CAS currently without a specific single gene diagnosis.

It is likely that inherited variants will account for a sizeable portion of CAS, but elucidation of these variants will require large cohorts coupled with deep phenotyping of family members. Interestingly, many families report a family history of speech difficulties, which might be explained by inherited variants that exhibit variable expressivity and phenotypic heterogeneity due to variability in the genetic background, similar to multi-hit models in other neurodevelopmental disorders [34].

The fact that many children with CAS exhibit comorbidities with ASD and ADHD suggests an additional genetic overlap with these conditions, which are mostly attributed to polygenic aetiology, as is the case for ASD [35]. A limitation in this nascent field of speech genetics is the lack of available population-based cohorts with both high quality genetic and phenotyping data. There is a concerted effort by the address this issue via the GenLang consortia (https://www.genlang.org); yet, to date, the cohorts in GenLang typically include language and literacy data, with recent fruitful GWAS publications identifying loci associated with language and literacy traits [36–38], but do not include speech-specific data.

One remaining challenge for the field is the lack of clinical variables which robustly predict who will have a monogenic cause or polygenic contributions (accepting that for monogenic diseases there may be modifier genetic contributions). From existing data, there is some evidence that individuals with any degree of cognitive impairment, or those more likely to have co-morbid language and motor diagnoses, have a greater likelihood of monogenic disease (Fig. 1c). However, it is clear that larger cohorts of individuals with CAS are required to provide adequate power to generate accurate genetic diagnostic prediction models to confirm these findings.

## Conclusions

After almost two decades with only one established gene for CAS, over 30 new genes of relevance have been identified in the past three years. Critically, about one third of children sequenced have received a molecular genetic diagnosis for their CAS, supporting implementation in clinical testing alongside other neurodevelopmental disorders. Around half of the candidate genes identified are currently supported by medium to strong evidence supporting the association between the gene and CAS. Almost all genes identified were previously described to cause neurodevelopmental conditions including ID, ASD and epilepsy. Hence the phenotypic spectrum of these conditions has been expanded to include individuals with a distinctly milder presentation of primary speech disorder. Whilst there is genetic heterogeneity, the genes coalesce on a small number of biological pathways, largely involved in chromatin remodelling or transcriptional regulation, providing new targets for precision medicines. Although genetic diagnoses in CAS to date have largely been de novo high impact variants in neurodevelopmental genes, the genetic architecture of CAS is likely to also encompass polygenic inheritance of common variants and rare inherited variants with incomplete penetrance and variable expressivity.

## Data Availability

Data for this review was collated from the three manuscripts meeting inclusion criteria (Kaspi et al., 2023; Hildebrand et al., 2020; Eising et al., 2020), or from additional manuscripts providing further supporting evidence of the association between apraxia of speech (phenotype) and specific genotypes which are all cited in the reference list. Hence all data is publicly available and replication possible using methods from the review.
